# Cumulative Query Method for Influenza Surveillance Using Search Engine Data

**DOI:** 10.2196/jmir.3680

**Published:** 2014-12-16

**Authors:** Dong-Woo Seo, Min-Woo Jo, Chang Hwan Sohn, Soo-Yong Shin, JaeHo Lee, Maengsoo Yu, Won Young Kim, Kyoung Soo Lim, Sang-Il Lee

**Affiliations:** ^1^Asan Medical CenterDepartment of Emergency MedicineUniversity of Ulsan, College of MedicineSeoulRepublic Of Korea; ^2^Asan Medical CenterDepartment of Preventive MedicineUniversity of Ulsan, College of MedicineSeoulRepublic Of Korea; ^3^Asan Medical CenterDepartment of Biomedical InformaticsSeoulRepublic Of Korea; ^4^Brigham and Women's HospitalDivision of General Medicine and Primary CareBoston, MAUnited States; ^5^Harvard Medical SchoolBoston, MAUnited States; ^6^Daum CommunicationsSearch Development UnitSeoulRepublic Of Korea

**Keywords:** syndromic surveillance system, influenza, influenza-like illness, Google Flu Trends, Internet search, query

## Abstract

**Background:**

Internet search queries have become an important data source in syndromic surveillance system. However, there is currently no syndromic surveillance system using Internet search query data in South Korea.

**Objectives:**

The objective of this study was to examine correlations between our cumulative query method and national influenza surveillance data.

**Methods:**

Our study was based on the local search engine, Daum (approximately 25% market share), and influenza-like illness (ILI) data from the Korea Centers for Disease Control and Prevention. A quota sampling survey was conducted with 200 participants to obtain popular queries. We divided the study period into two sets: Set 1 (the 2009/10 epidemiological year for development set 1 and 2010/11 for validation set 1) and Set 2 (2010/11 for development Set 2 and 2011/12 for validation Set 2). Pearson’s correlation coefficients were calculated between the Daum data and the ILI data for the development set. We selected the combined queries for which the correlation coefficients were .7 or higher and listed them in descending order. Then, we created a cumulative query method *n* representing the number of cumulative combined queries in descending order of the correlation coefficient.

**Results:**

In validation set 1, 13 cumulative query methods were applied, and 8 had higher correlation coefficients (min=.916, max=.943) than that of the highest single combined query. Further, 11 of 13 cumulative query methods had an *r* value of ≥.7, but 4 of 13 combined queries had an *r* value of ≥.7. In validation set 2, 8 of 15 cumulative query methods showed higher correlation coefficients (min=.975, max=.987) than that of the highest single combined query. All 15 cumulative query methods had an *r* value of ≥.7, but 6 of 15 combined queries had an *r* value of ≥.7.

**Conclusions:**

Cumulative query method showed relatively higher correlation with national influenza surveillance data than combined queries in the development and validation set.

##  Introduction

Syndromic surveillance may alert public health care providers in the early phases of an outbreak, allowing them to decrease morbidity and mortality resulting from the outbreak [1-5]. Syndromic surveillance is defined as the real-time or near real-time collection, analysis, interpretation, and dissemination of health-related data to enable the early identification of the impact of potential human or veterinary public health threats that require effective public health action [1,3]. The 2009 H1N1 influenza pandemic highlighted the need for syndromic surveillance to inform policy and plan for effective responses.

Because conventional syndromic surveillance of indicators such as influenza-like illness (ILI) depends on case reporting to report disease activity, time delays in reporting and case confirmation can interfere with the early detection of outbreaks or increases in influenza cases in the community. Thus, researchers have been investigating alternative data sources for the detection of outbreaks. For example, over-the-counter sales of medications and school absenteeism data have been used for earlier detection of outbreaks [6-12].

Internet search queries have become an important data source in recent years [13-22]. Internet search engines allow billions of people to have instant access to a vast amount of information online. New syndromic surveillance sources, such as Google Flu Trends (GFT), provide the potential to identify influenza outbreaks in real time [23]. Several studies have reported that GFT is highly correlated with conventional ILI surveillance data [23-28]. GFT has now been applied in many countries, but neither GFT nor other search query-based tools for disease surveillance are available in South Korea [24,27-30]. Generally, Google’s market share is dominant in the countries where GFT is available [24-29,31], but not in South Korea [32]. Studies using Google Trends for influenza surveillance show that it can be used as a complementary source of data but that its performance is insufficient for use as a model for prediction [33,34]. It is difficult to find queries that show high correlations for consecutive years because Internet searching behavior may change over time [33,34]. To reduce the effects from changes in search queries, we used a combination of queries and cumulation of combined queries from the search engine Daum. Daum is the second largest Web portal service provider in daily visits in South Korea (approximately 25% of the market share) [32,35]. Daum offers many Internet services to Web users, including email, messaging service, forums, shopping, and news. The main language is Korean.

In South Korea, influenza is generally seasonal, with most activity occurring during winter. The 2009/10 epidemiological year, called the Influenza A (H1N1) pandemic period, was an exceptional situation (see Multimedia Appendix 1). The primary objective of this study was to examine correlations between our cumulative query method and national influenza surveillance data.

## Methods

### Source of Data

#### Study Period

The study period was September 6, 2009 (week 36), through September 1, 2012 (week 34)—156 weeks of data for 3 consecutive epidemiological years. We divided the study period into two sets: Set 1 (the 2009/10 epidemiological year for development set 1 and 2010/11 for validation set 1) and Set 2 (2010/11 for development set 2 and 2011/12 for validation set 2).

#### Collection of Influenza-Like Illness Data

We collected the ILI data from the Korea Centers for Disease Control and Prevention (KCDC) as a gold standard. KCDC ILI data were available from the KCDC website; we downloaded the ILI data for the study period from this site [36]. A KCDC case of ILI was defined as a person with a fever of 38°C with a cough and/or a sore throat [36]. ILI surveillance consisted of 850 sentinel clinics in South Korea, and the clinics reported weekly percentages of outpatients who met the case definition of ILI [36].

#### Survey for Obtaining Queries

To obtain population search queries related to influenza, we conducted a survey from quota sampling based on sex and age in September 2012. The quotas were based on address of resident registry, age, and sex. There were five quota groups by age: 20-29 years, 30-39 years, 40-49 years, 50-59 years, 60 years or older. Half of each quota group were female. We randomly selected the addresses from the residence registry in Seoul, and then if interviewees living at the address of residence registry met the criteria, we included the oldest interviewee. We then conducted face-to-face interviews. The survey included searching history for influenza and typed queries. The survey was performed anonymously. A KCDC definition of ILI was a person with a fever (발열 in Korean) of 38°C with a cough (기침) and/or a sore throat (인후통). These three queries from the definitions of ILI were included in the queries for the following operations, regardless of the survey result. In the case of queries originally submitted in English only, we translated them to Korean and added them as new queries.

#### Combination of Queries

We believe that people typically search for things of interest on the Internet using one or more queries at a time. To reflect people’s searching behavior and include as many queries as possible, we used a combination of queries. Queries from the survey results and the definition of KCDC ILI were divided into groups as follows: query group 1 consisted of queries specific to influenza (eg, “H1N1”, “Influenza”), and query group 2 contained queries not specific to influenza (eg, “Treatment”, “Symptom”). Then, we combined query groups 1 and 2. Combined queries consisted of query group 1 alone and a combination of query groups 1 and 2 (eg, “H1N1”, “H1N1 Treatment”, “H1N1 Symptom”, “Influenza”, “Influenza Treatment”, “Influenza Symptom”).

#### Collection of Data from Search Engine

We sent the combined queries and the queries that belonged to query group 1 (because these queries were searchable by themselves) to Daum and received proportional data in weekly form. Proportional data for these combined queries were extracted from the Daum search engine during development sets 1 and 2. Proportional data from the Daum search engine were calculated by dividing the number of each combined query by the total number of search queries for 1 week.

### Data Analysis

#### Creating Cumulative Query Methods and Data Analysis

Pearson’s correlation coefficients were calculated between the Daum data for the combined queries and the KCDC ILI data in development sets 1 and 2. We selected the combined queries for which the correlation coefficients were .7 or higher and listed them in descending order. To see the change of correlation coefficients over time, we also calculated correlation coefficients of the combined queries in subsequent epidemiological years. We then created a cumulative query method *n* representing the number of cumulative combined queries in descending order of the correlation coefficient. For example, cumulative query method 4 consisted of a summation of the proportional data from the 1st, 2nd, 3rd, and 4th highest combined queries on the correlation coefficient list. In validation sets 1 and 2, Pearson’s correlation coefficients were calculated between the cumulative query method *n* and the KCDC ILI data. Specifically in validation set 2, we analyzed the cumulative query methods from development set 2 as well as development set 1. Useful cumulative query methods in the validation sets were defined as having higher correlation coefficienst than the highest correlation coefficient of a single combined query in the same development set. Analysis was performed using IBM SPSS Statistics software, version 20. Significance was set at *P*<.05.

#### Institutional Review Board

This study was approved by the Institutional Review Board of Asan Medical Center (Seoul, Korea).

## Results

### Survey for Obtaining Queries

We contacted 322 people and included 200 participants older than 20 years who lived in Seoul, Korea. Over a quarter (56/200, 28%) answered “Yes” to the question of searching history for influenza and provided search queries (Table 1).

**Table 1:** Results of the survey.

Raw data	English translation	Frequency (%)
신종	New	1 (1.8)
신종플루	New flu^a^	23 (41.1)
신종플루 증상	New flu symptom	1 (1.8)
신종플루 증세	New flu sign	1 (1.8)
신종플루, 독감	New flu, bad cold	2 (3.6)
신종플루, 목아픔	New flu, neck pain	1 (1.8)
신종플루, 백신, Tamiflu	New flu, vaccine, Tamiflu (English)^b^	1 (1.8)
신종플루, 신플 증상	New flu, new flu (abbr.)^c^ symptom	1 (1.8)
신종플루, 인플루엔자, H1N1, PCR	New flu, influenza, H1N1 (English)^b^, PCR^d^ (English)^b^	1 (1.8)
신종플루, 조류독감	New flu, bird flu	1 (1.8)
신종플루의 치료, 합병증	New flu, treatment, complication	1 (1.8)
신종플루증상	New flu symptom	1 (1.8)
신종플루증세, 예방, 마스크	New flu sign, prevention, mask	1 (1.8)
신플증상	New flu (abbr.)^c^ symptom	1 (1.8)
열, 기침	Fever, cough	1 (1.8)
유행성독감, influenza	Epidemic bad cold, influenza (English)^b^	1 (1.8)
인플루엔자	Influenza	7 (12.5)
인플루엔자, 신종독감, 신종 플루	Influenza, new bad cold, new flu	1 (1.8)
인플루엔자, 조류독감	Influenza, bird flu	1 (1.8)
인플루엔자, 조류독감, 돼지독감, 신종플루	Influenza, bird flu, swine flu, new flu	1 (1.8)
조류독감	Bird flu	5 (8.9)
조류독감, 사망	Bird flu, decease	1 (1.8)
증상, 목통증	Symptom, throat pain	1 (1.8)
Total		200 (100.0)

^a^Since the Influenza A (H1N1) pandemic period, media began to use “New flu (신종플루)” to distinguish the H1N1 influenza and previous influenzas in Korea. In 2010, KCDC announced that the official term was “Influenza (인플루엔자)”. But “New flu (신종플루)” and “Bad cold (독감)” are still more popular terms than “Flu (플루)” or “Influenza (인플루엔자)” in Korea. “Bad cold (독감)” in Korean has two meanings: one is influenza and the other, a severe common cold.

^b^The query was originally submitted in English.

^c^Abbreviation: “New flu (abbr.) (신플)” is the abbreviation of “New flu (신종플루)” in Korean.

^d^PCR: polymerase chain reaction.

### Combination of Queries From the Survey

Query group 1 contained 14 queries that were specific to influenza, and query group 2 had 14 queries that were not specific to influenza (Table 2). A total of 210 combined queries were submitted to Daum. Full data of combined queries are presented in Multimedia Appendix 2.

**Table 2:** Query groups 1 and 2 from the survey results and the KCDC definition of ILI^a^.

Query group 1	Query group 2
Flu	Vaccine
New flu	Prevention
New flu (abbr.)^b^	Mask
Influenza	Symptom
Influenza (English)^c^	Sign
New influenza	Cough
Bad cold^d^	Fever
New bad cold	Neck pain
Epidemic bad cold	Sore throat
H1N1 (English)^c^	Throat pain
Bird flu	PCR (English)^c,e^
Swine flu	Treatment
Tamiflu	Complication
Tamiflu (English)^c^	Decease

^a^Query group 1 consisted of queries specific to or related to influenza. Query group 2 contained queries not specific to influenza.

^b^Abbreviation.

^c^The query was originally submitted in English.

^d^“Bad cold (독감)” in Korean has two meanings: one is influenza and the other, a severe common cold. “Flu” in query group 1 is “플루” which is the English pronunciation written in Korean. In Korea, “Bad cold (독감)” is a more popular term than “Flu (플루)” or “Influenza (인플루엔자)”.

^e^PCR: polymerase chain reaction.

### Collection of Data From Search Engine

**Figure 1:**
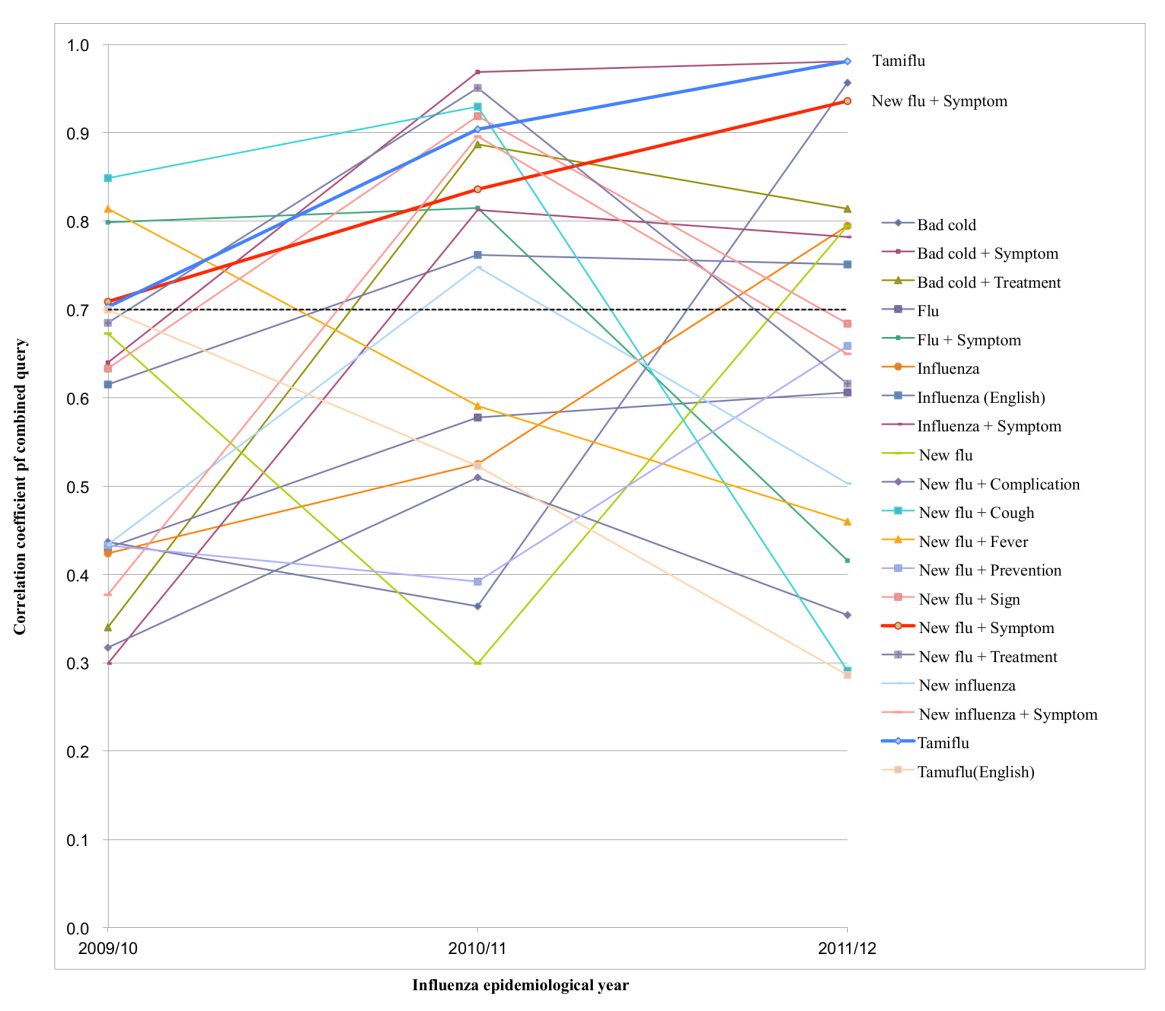
Plot of combined queries that consecutively show correlation coefficient (P<.05) (only “Tamiflu” and “New flu + Symptom” showed r values greater than .7 for 3 consecutive years).

Correlation analysis was performed between the Daum data for combined queries and the KCDC ILI data in development sets 1 and 2 (Table 3). In development set 1, “New flu (abbr.)” had the highest correlation coefficient (*r*=.894, *P*<.001), and 13 combined queries had correlation coefficient *r* values of ≥.7. Among these 13 combined queries, the number of the combined queries that had correlation coefficient *r* values of ≥.7 was reduced to 4 in validation set 1 and to 2 in validation set 2. In development set 2, “Bad cold + Symptom” had the highest correlation coefficient (*r*=.969, *P*<.001), and a total of 15 combined queries had an *r* value of ≥.7. Among these 15 combined queries, the number of the combined queries that had correlation coefficient *r* values of ≥.7 was reduced to 6 in validation set 2. Only “Tamiflu” and “New flu + Symptom” showed correlation coefficients *r* values of ≥.7 for 3 consecutive years (Figure 1). The change of correlation coefficients for all combined queries over time are presented in Multimedia Appendix 2.

**Table 3:** Correlation analysis between the Daum data for combined queries and the KCDC ILI data in development sets 1 and 2.

Order	Combined query	Correlation coefficient			Combined query	Correlation coefficient	
		Development set 1 (2009/10)	Validation set 1 (2010/11)	Validation set 2 (2011/12)		Development set 2 (2010/11)	Validation set 2 (2011/12)
1	New flu (abbr.)^a^	.894^b^	.622^b^	^c^	Bad cold + Symptom	.969^b^	.981^b^
2	Flu + Vaccine	.871^b^	-.062^d^	-.157^e^	New flu + Treatment	.951^b^	.616^b^
3	New flu + Cough	.849^b^	.930^b^	.291^b^	New flu + Cough	.930^b^	.291^b^
4	New flu + Fever	.814^b^	.591^b^	.460^b^	New flu + Sign	.919^b^	.684^b^
5	Tamiflu + Vaccine	.805^b^	-.062^c^	^c^	Tamiflu	.904^b^	.981^b^
6	Tamiflu + Symptom	.800^b^	^c^	^c^	New influenza + Symptom	.896^b^	.650^b^
7	Flu + Symptom	.799^b^	.815^b^	.416^b^	Bad cold + Treatment	.887^b^	.814^b^
8	H1N1 + Symptom	.791^b^	^c^	^c^	Swine flu + Symptom	.877^b^	.005^e^
9	New flu + Sore throat	.738^b^	.504^b^	^c^	New flu + Symptom	.836^b^	.936^b^
10	New flu (abbr.)^a^ + Vaccine	.713^b^	^c^	^c^	Flu + Symptom	.815^b^	.416^b^
11	New flu + Symptom	.709^b^	.836^b^	.936^b^	Influenza + Symptom	.813^b^	.782^b^
12	Tamiflu	.703^b^	.904^b^	.981^b^	Influenza (English)^g^	.762^b^	.751^b^
13	Tamiflu (English)^g^	.700b^b,h^	.523^b^	.286^b^	New influenza	.748^b^	.503^b^
14					Bird flu + Symptom	.747^b^	.005^f^
15					Bird flu	.709^b,h^	.136^i^

^a^abbr.: abbreviation

^b^ *P*<.05.

^c^Correlation cannot be computed because it has a constant value in that period (see Multimedia Appendix 2).

^d^ *P=*.66.

^e^ *P=*.27.

^f^ *P=*.98.

^g^The query was originally submitted in English.

^h^We selected the combined queries for which the correlation coefficients were ≥.7 and listed them in descending order.

^i^ *P=*.34.

###  Creating Cumulative Query Methods

**Figure 2:**
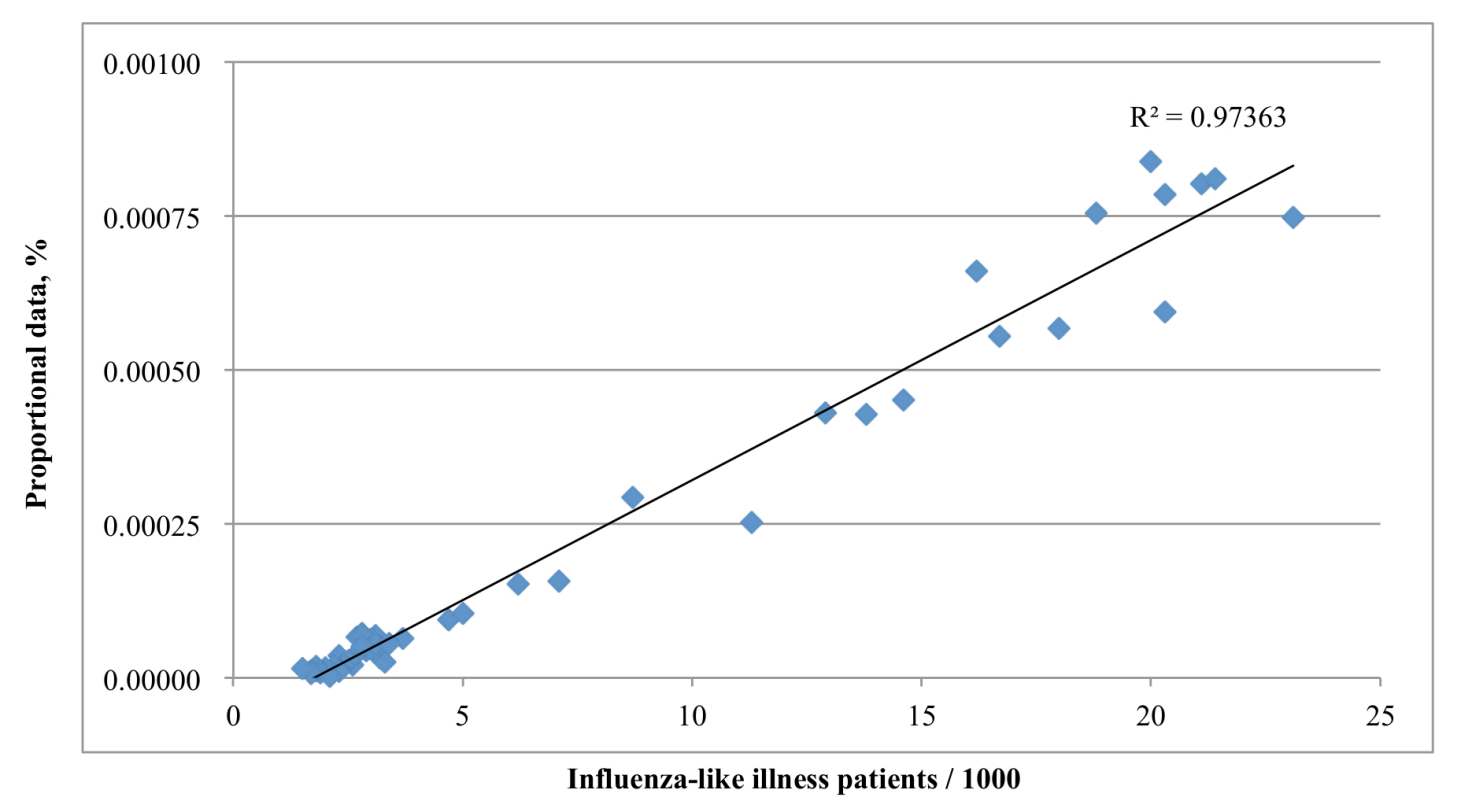
Scatter plot between the KCDC ILI and cumulative query model 5 in validation set 2.

A total of 13 cumulative query methods were created in development set 1 (see Table 4). In validation set 1, cumulative query methods 7, 8, 9, and 10 showed the highest correlation coefficients (*r*=.943, *P*<.001; see Multimedia Appendix 3). Eight of the 13 cumulative query methods were useful, which was defined as having higher correlation coefficients than the highest correlation coefficient of a single combined query in development set 1 (min=.916, max=.943). But only three of the cumulative query methods from development set 1 were useful in validation set 2 (min=.935, max=.953). The correlation did not increase by adding queries in cumulative query method 5, 6, 8, 9, 10, and 13 in validation set 1. In validation set 2, cumulative query method 5 from development set 2 had the highest correlation coefficient (*r*=.987, *P*<.001; see Figure 2 and Multimedia Appendix 3). Eight of the 15 cumulative query methods from development set 2 were useful (min=.975, max=.987). The correlation did not increase by adding queries in cumulative query method 3, 4, 7, 8, 10, 12, and 14 in validation set 2. Scatter plots between the KCDC ILI and other useful cumulative query methods are presented in Multimedia Appendix 4. Cumulative query methods for influenza virologic data are presented in Multimedia Appendix 5.

**Figure 3:**
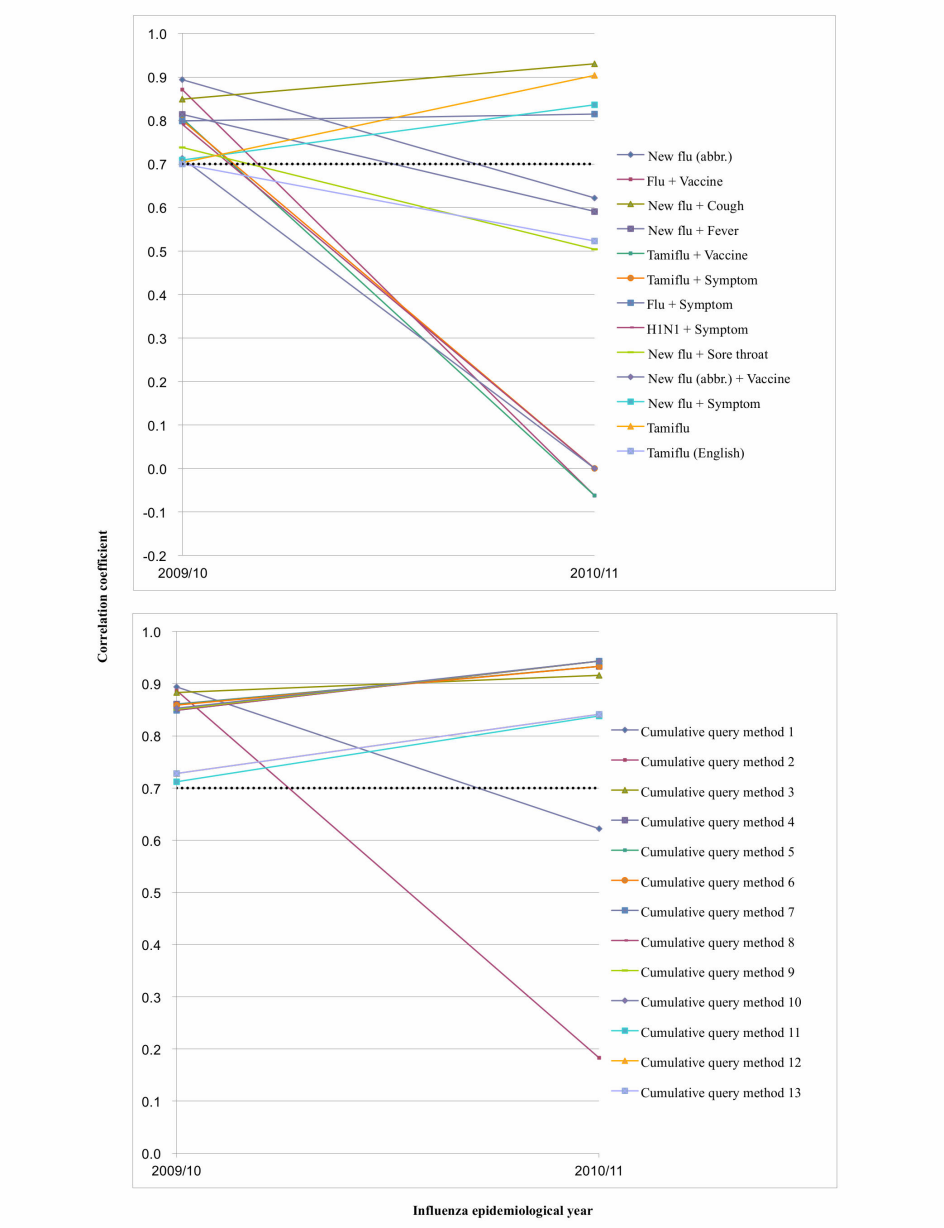
Plot of combined queries for which the correlation coefficients were .7 or higher and cumulative query methods of set 1.

**Figure 4:**
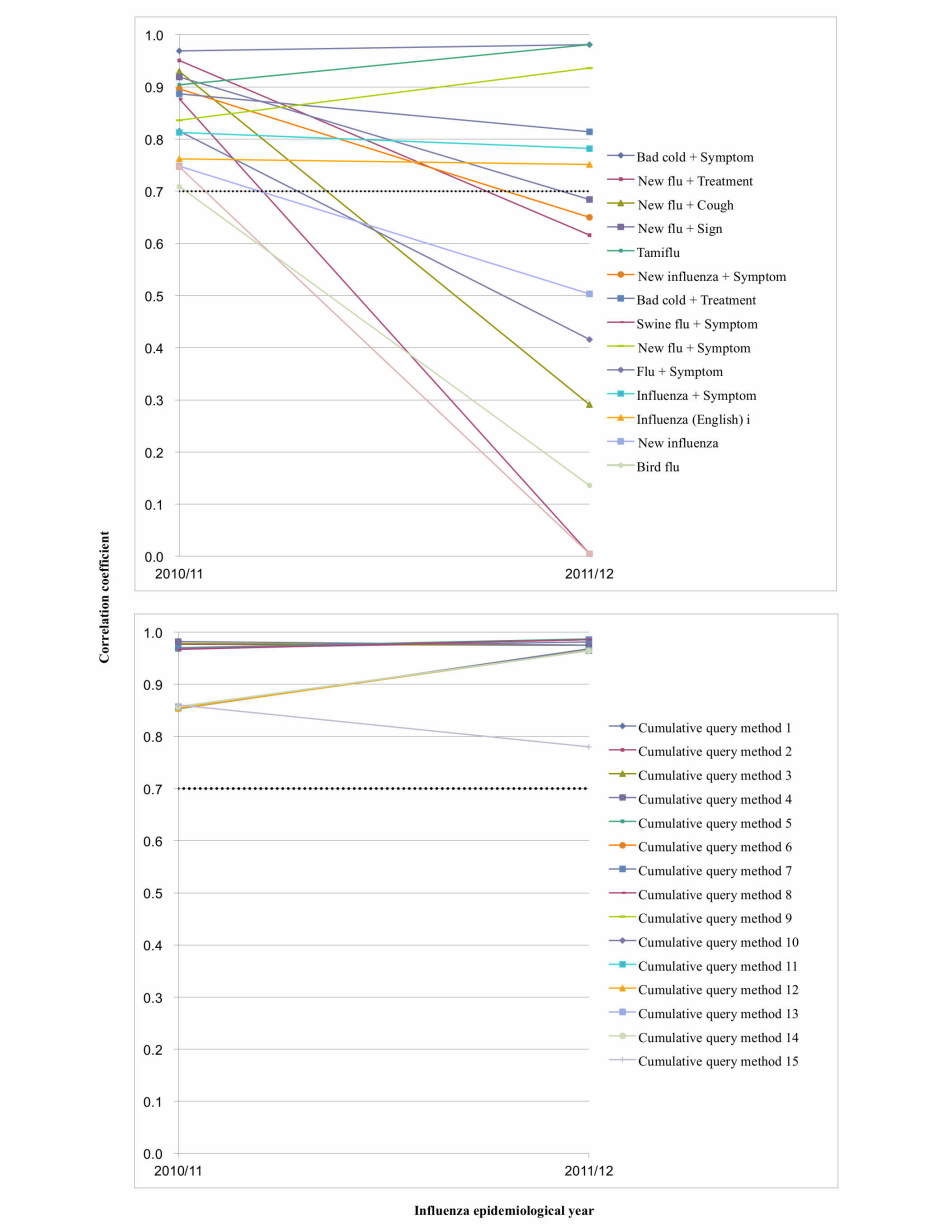
Plot of combined queries for which the correlation coefficients were .7 or higher and cumulative query methods of set 2.

In each development set, cumulative query methods had a higher correlation coefficient than combined queries (see Tables 5 and 6). After 1 year, 11 of 13 cumulative query methods had an *r* value of ≥.7, but 4 of 13 combined queries had an *r* value of ≥.7 in validation set 1 (see Table 5 and Figure 3). All 15 cumulative query methods had an *r* value of ≥.7, but 6 of 15 combined queries had an *r* value of ≥.7 in validation set 2 (see Table 6 and Figure 4).

**Table 4:** Correlation coefficients of cumulative query method *n* in each validation set^a^.

Cumulative query method	Correlation coefficient in validation set 1	Correlation coefficient in validation set 2 from development set 1	Correlation coefficient in validation set 2 from development set 2
1	.622^b^	^c^	.981^b,d^
2	.183^e^	-.157^f^	.975^b,d^
3	.916^b,d^	.092^g^	.975^b,d^
4	.933^b,d^	.467^b^	.975^b,d^
5	.933^b,d^	.467^b^	.987^b,d^
6	.933^b,d^	.467^b^	.986^b,d^
7	.943^b,d^	.486^b^	.986^b,d^
8	.943^b,d^	.486^b^	.986^b,d^
9	.943^b,d^	.486^b^	.968^b^
10	.943^b,d^	.486^b^	.968^b^
11	.838^b^	.935^b,d^	.965^b^
12	.841^b^	.953^b,d^	.965^b^
13	.841^b^	.953^b,d^	.964^b^
14	Not applicable	Not applicable	.964^b^
15	Not applicable	Not applicable	.780^b^

^a^We selected the combined queries for which the correlation coefficients were ≥.7 and listed them in descending order. We then created a cumulative query method *n* representing the number of cumulative combined queries in descending order of the correlation coefficients.

^b^ *P*<.05.

^c^Correlation of cumulative query method 1 in validation set 2 from development set 1 cannot be computed because it has a constant value in that period (see Multimedia Appendix 2).

^d^Useful cumulative query method in the validation set was defined as having higher correlation coefficient than the highest correlation coefficient of a single combined query in the same development set.

^e^ *P=*.20.

^f^ *P=*.27.

^g^ *P=*.52.

**Table 5:** Correlation coefficients of combined queries for which the correlation coefficients were ≥.7 and cumulative query methods in set 1.

Cumulative query method from development set 1 (2009/10)	Correlation coefficient		Combined query from development set 1 (2009/10)	Correlation coefficient	
	2009/10	2010/11		2009/10	2010/11
1	.894	.622	New flu (abbr.)^a^	.894	.622
2	.887	.183	Flu + Vaccine	.871	-.062
3	.883	.916	New flu + Cough	.849	.93
4	.861	.933	New flu + Fever	.814	.591
5	.86	.933	Tamiflu + Vaccine	.805	-.062
6	.859	.933	Tamiflu + Symptom	.8	.^b^
7	.849	.943	Flu + Symptom	.799	.815
8	.849	.943	H1N1 + Symptom	.791	.^b^
9	.851	.943	New flu + Sore throat	.738	.504
10	.853	.943	New flu (abbr.)^a^ + Vaccine	.713	.^b^
11	.712	.838	New flu + Symptom	.709	.836
12	.728	.841	Tamiflu	.703	.904
13	.728	.841	Tamiflu (English)^c^	.7	.523

^a^abbr.: abbreviation

^b^Correlation cannot be computed because it has a constant value in that period (see Multimedia Appendix 2).

^c^The query was originally submitted in English.

**Table 6:** Correlation coefficients of combined queries for which the correlation coefficients were ≥.7 and cumulative query methods in set 2.

Cumulative query method from development set 2 (2010/11)	Correlation coefficient		Combined query from development set 2 (2010/11)	Correlation coefficient	
	2010/11	2011/12		2010/11	2011/12
1	.969	.981	Bad cold + Symptom	.969	.981
2	.977	.975	New flu + Treatment	.951	.616
3	.978	.975	New flu + Cough	.93	.291
4	.982	.975	New flu + Sign	.919	.684
5	.97	.987	Tamiflu	.904	.981
6	.968	.986	New influenza + Symptom	.896	.65
7	.969	.986	Bad cold + Treatment	.887	.814
8	.967	.986	Swine flu + Symptom	.877	.005
9	.853	.968	New flu + Symptom	.836	.936
10	.853	.968	Flu + Symptom	.815	.416
11	.854	.965	Influenza + Symptom	.813	.782
12	.854	.965	Influenza (English)^a^	.762	.751
13	.857	.964	New influenza	.748	.503
14	.857	.964	Bird flu + Symptom	.747	.005
15	.86	.78	Bird flu	.709	.136

^a^The query was originally submitted in English.

## Discussion

### Principal Findings

In this study, the cumulative query method showed relatively higher correlation with national influenza surveillance data than combined queries in the development and validation set.

Many people use Internet searches for health information before visiting a doctor [18,20,23,33]. Hence, search query trends can reflect actual disease progression earlier than conventional surveillance. Queries used prior to this study only reflected the authors’ opinions [13] or were obtained from databases [13,14,23,37]. To obtain population search queries, we carried out a study survey.

Search queries may vary from country to country. In Korea, “Bad cold (독감)” in Korean has two meanings: one is influenza and the other, a severe common cold. Since the 2009/10 epidemiological season, the Influenza A (H1N1) pandemic period, the media began to use “New flu (신종플루)” in order to distinguish H1N1 influenza and previous influenzas. In 2010, KCDC announced that the official term was “Influenza (인플루엔자)” [36]. But “New flu (신종플루)” and “Bad cold (독감)” are still more popular terms than “Flu (플루)” or “Influenza (인플루엔자)” in Korea (Table 1).

For the 2009/10 epidemiological year (development set 1), 13 combined queries had correlation coefficient *r* values ≥.7. However, only 4 of these combined queries (“New flu + Cough”, “Flu + Symptom”, “New flu + Symptom”, and “Tamiflu”) had correlation coefficient *r* values ≥.7 in the 2010/11 epidemiological year (validation set 1) (Table 3). But 11 of 13 cumulative query methods had an *r* value of ≥.7 in validation set 1 (see Table 5 and Figure 3). Among 15 combined queries of development set 2, the number of the combined queries that had correlation coefficient *r* values of ≥.7 reduced to 6 in validation set 2. But all 15 cumulative query methods had an *r* value of ≥.7 in validation set 2. We think that the cumulative query method is more robust with time, and this factor is helpful for improving surveillance performance using search queries. Since Internet searching behavior may change over time, this could have affected the performance of Web query-based surveillance model [31]. In this study, 20 out of 210 combined queries had correlation coefficients for all 3 years. And only “Tamiflu” and “New flu + Symptom” showed correlation coefficients *r* values of ≥.7 for 3 consecutive years (see Figure 1 and Multimedia Appendix 2). Recently, a study using Google Trends for influenza surveillance showed that Google Trends can be used as a complementary source of data [33]; however, its performance is insufficient for use as a model for prediction because its maximum correlation coefficient was .82 for only one query, “Fever”, in 2009, and the coefficient decreased to .64 in 2011 [33].

It is difficult to predict the change of search queries in the future. To reduce the effects from changes in search queries, we used a combination of queries and cumulation of combined queries to construct our method. Additionally, the method we wanted to develop was meaningful only when the cumulative query method had a higher correlation coefficient than the highest single combined query. In each validation set, 8 useful cumulative query methods were developed. The useful cumulative query methods in each validation set had a high correlation coefficient (Table 4). In validation set 2, the range of correlation coefficients of the useful cumulative query methods was from .975 to .987. These values are similar to or higher than those reported elsewhere [13,14,23,26,27,29,31]. In Europe, correlation coefficients of .716 to .940 were reported for GFT [27], and coefficients of .82 to .99 were reported in the United States [23,26,31]. In the 2009/10 epidemiological year, called the Influenza A (H1N1) pandemic period, the proportional data of queries were likely to have been different compared to the other epidemiological year. It might affect performance of cumulative query methods in set 1. The performance of the cumulative query method in set 1 was decreased with time (Table 4). It is thought to be related to the changes of queries (see Table 3). For some cumulative query methods, the correlation did not increase by adding queries. The added query did not give extra value in the cumulative query methods 6, 8, and 10 in validation set 1 (see Table 4 and Multimedia Appendix 2). Combined queries 6, 8, and 10 from development set 1 in validation set 1 have a constant value 0 (see Table 3 and Multimedia Appendix 2). The added queries were relatively too small compare to the previous queries in the cumulative query methods 5, 9, 10 in validation sets 1, 3, 4, 7, 8, 10, 12, and 14 in validation set 2 (see Table 4 and Multimedia Appendix 2).

We used proportional data from Daum, a non-dominant local search engine (approximately 25% of the market share) in South Korea [32]. Our cumulative query methods showed a strong correlation with KCDC ILI data. Generally, Google’s market share is dominant in countries where GFT is available [27,28]. Our study showed the possibility of developing a surveillance model using a non-dominant local search engine.

### Limitations

There are several limitations to this study. The survey of our study is not a representative sample. Because respondents were asked to provide typed queries without mention of the influenza pandemic of 2009/10, recent search queries were more likely to have been included in this study because the survey was conducted recently. This might affect performance of the cumulative query method. Further, the data from the influenza pandemic of 2009/10 might affect the outcome of this study. In this study, we did not combine queries from the same query group. Although important, the performance of using symptoms in the definition of KCDC ILI was not tested. The learning effect from the influenza pandemic of 2009/10, news reports, outbreak briefs, health information from the Internet, and changing search behavior stemming from the diffusion of smartphones might have affected the outcome of this study. We did not determine the extent to which these factors affected the searching behavior. More data for subsequent years are required in order to know the life of the cumulative query method.

### Conclusion

We presented a cumulative query method using search engine data. We conducted a survey to obtain population search queries. To reduce the effects from changes in search queries, we used a combination of queries and cumulation of combined queries. Our method showed high correlation with national influenza surveillance data in South Korea. However, to further our method, additional research is needed.

## Multimedia Appendix 1

Seasonality of influenza in South Korea.

## Multimedia Appendix 2

Full study data (proportional data from Daum are multiplied by 12 squares of 10).

## Multimedia Appendix 3

Scaled proportional data based on the two best cumulative query methods.

## Multimedia Appendix 4

Scatter plots between the KCDC ILI and other useful cumulative query methods.

## Multimedia Appendix 5

Cumulative query method for influenza virologic data.

## Abbreviations

GFT: Google Flu Trends
ILI: influenza-like illness
KCDC: Korea Centers for Disease Prevention and Control
PCR: polymerase chain reaction
