# Supplementary figures and images for: Cumulative Query Method for Influenza Surveillance Using Search Engine Data
Source: J Med Internet Res. 2014 Dec 16;16(12):e289. doi: 10.2196/jmir.3680 (PMC4275481; doi:10.2196/jmir.3680)

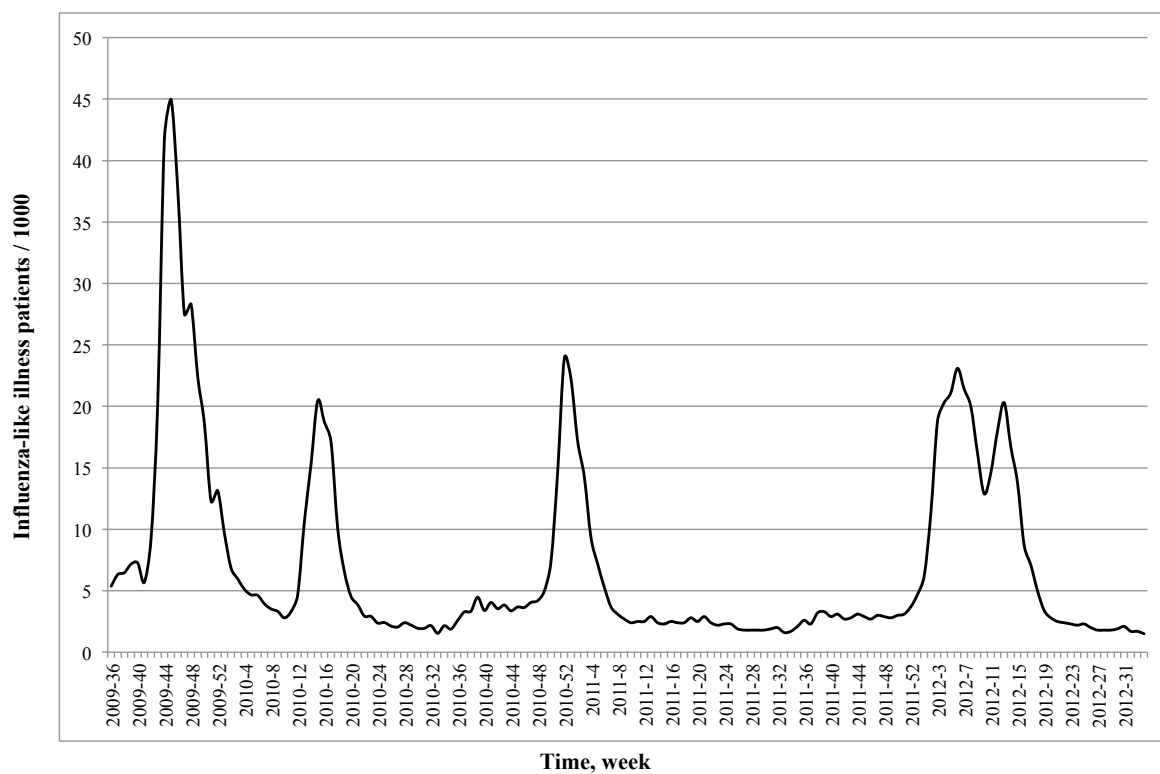

Supplement: Supplementary file 1 [file jmir_v16i12e289_app1.pdf]
