# Supplementary material for: Cumulative Query Method for Influenza Surveillance Using Search Engine Data
Source: J Med Internet Res. 2014 Dec 16;16(12):e289. doi: 10.2196/jmir.3680 (PMC4275481; doi:10.2196/jmir.3680)
Supplement: Supplementary file 3 [file jmir_v16i12e289_app3.pdf]

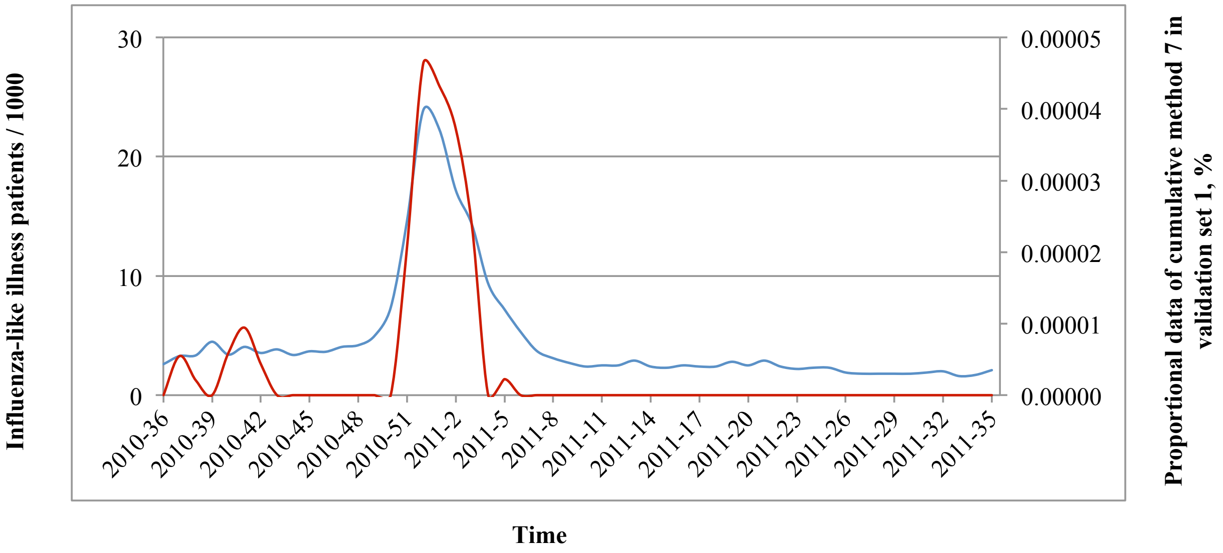
Influenza-like illness patients / 1000
Proportional data of cumulative method 7 in validation set 1, %
30
20
10
0
0.00005
0.00004
0.00003
0.00002
0.00001
0.00000
2010-36
2010-39
2010-42
2010-45
2010-48
2010-51
2011-2
2011-5
2011-8
2011-11
2011-14
2011-17
2011-20
2011-23
2011-26
2011-29
2011-32
2011-35
Time

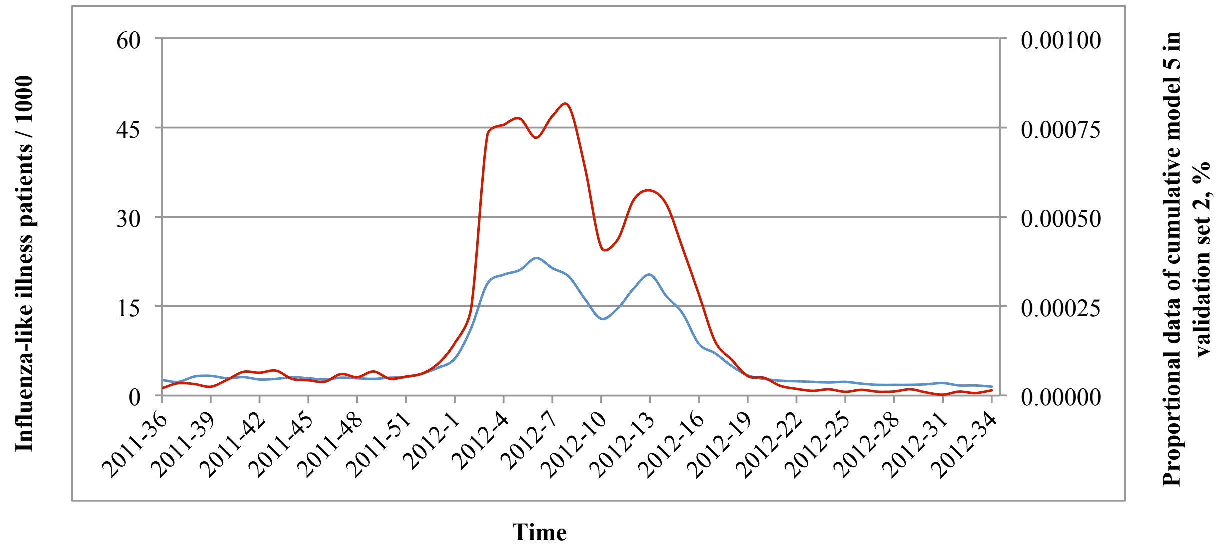
Influenza-like illness patients / 1000
Proportional data of cumulative model 5 in validation set 2, %
60
45
30
15
0
0.00100
0.00075
0.00050
0.00025
0.00000
2011-36
2011-39
2011-42
2011-45
2011-48
2011-51
2012-1
2012-4
2012-7
2012-10
2012-13
2012-16
2012-19
2012-22
2012-25
2012-28
2012-31
2012-34
Time
