# Supplementary material for: Cumulative Query Method for Influenza Surveillance Using Search Engine Data
Source: J Med Internet Res. 2014 Dec 16;16(12):e289. doi: 10.2196/jmir.3680 (PMC4275481; doi:10.2196/jmir.3680)
Supplement: Supplementary file 4 [file jmir_v16i12e289_app4.pdf]

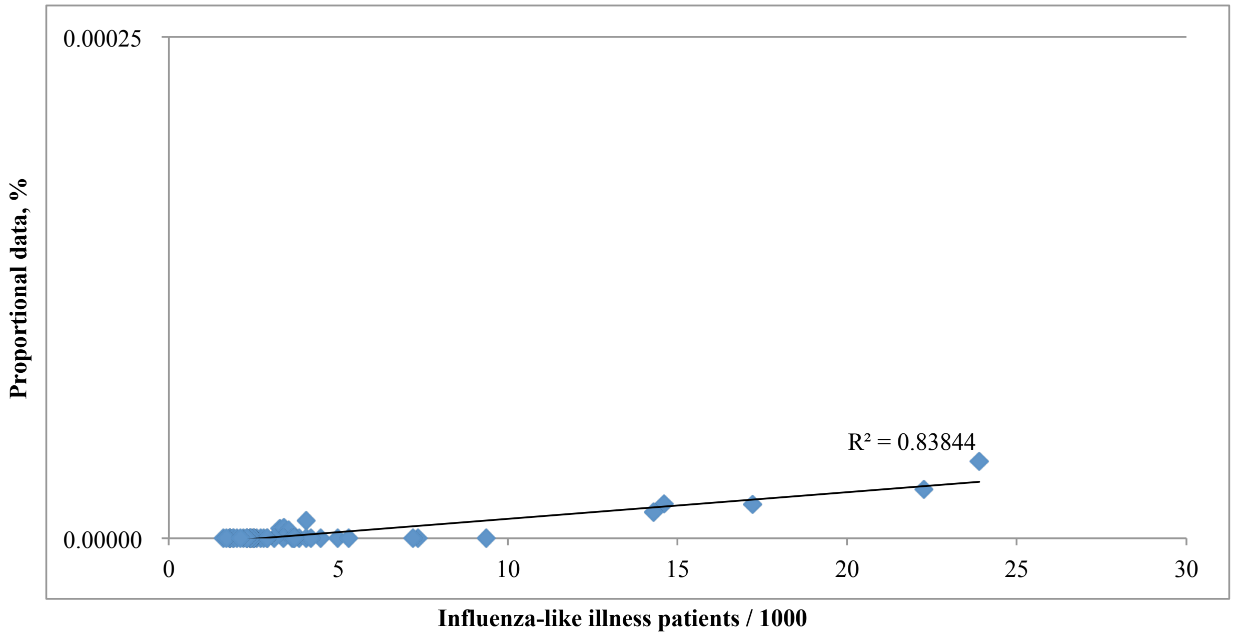

Cumulative query model 3 in validation set 1

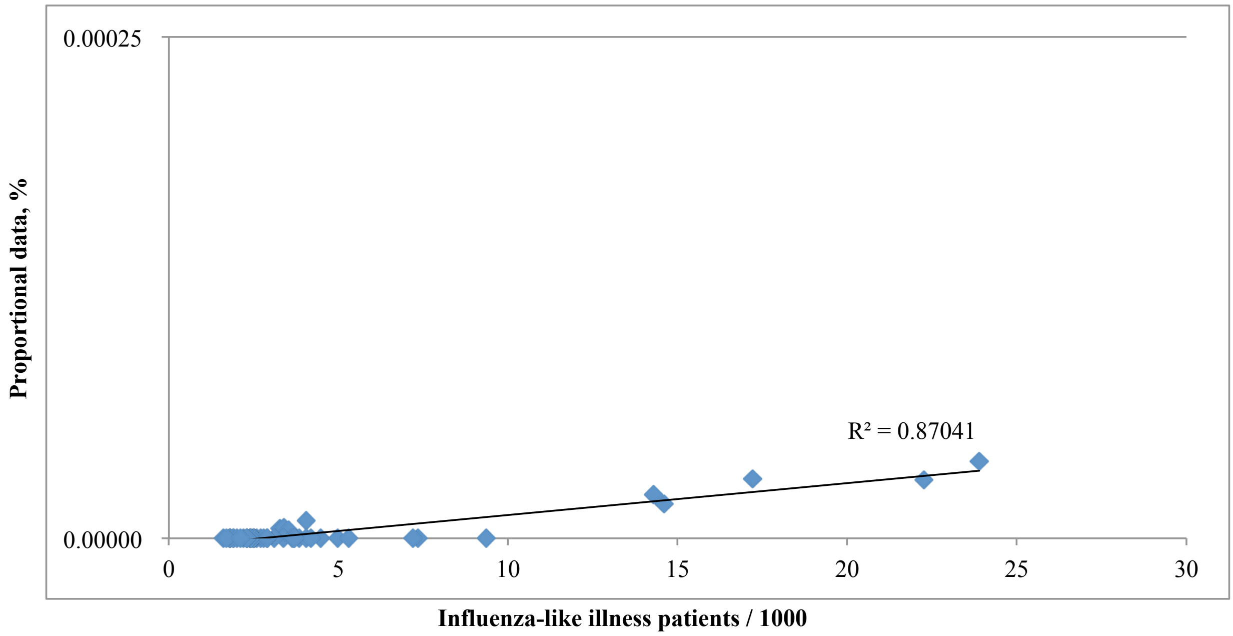

Cumulative query model 4 in validation set 1

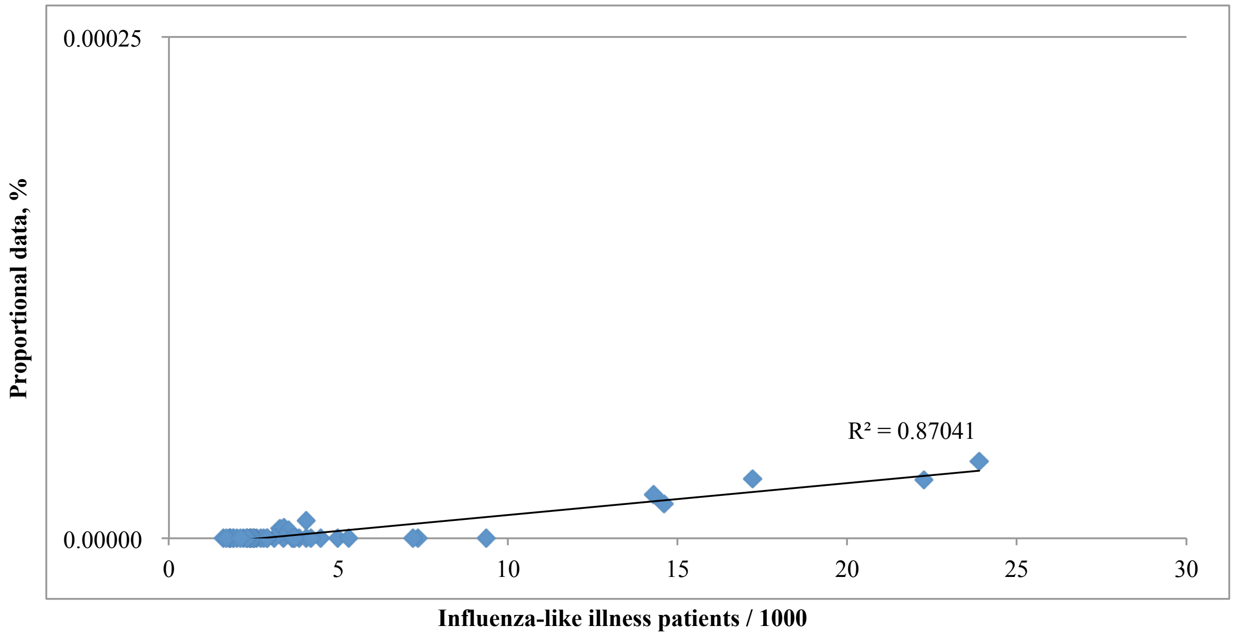

Cumulative query model 5 in validation set 1

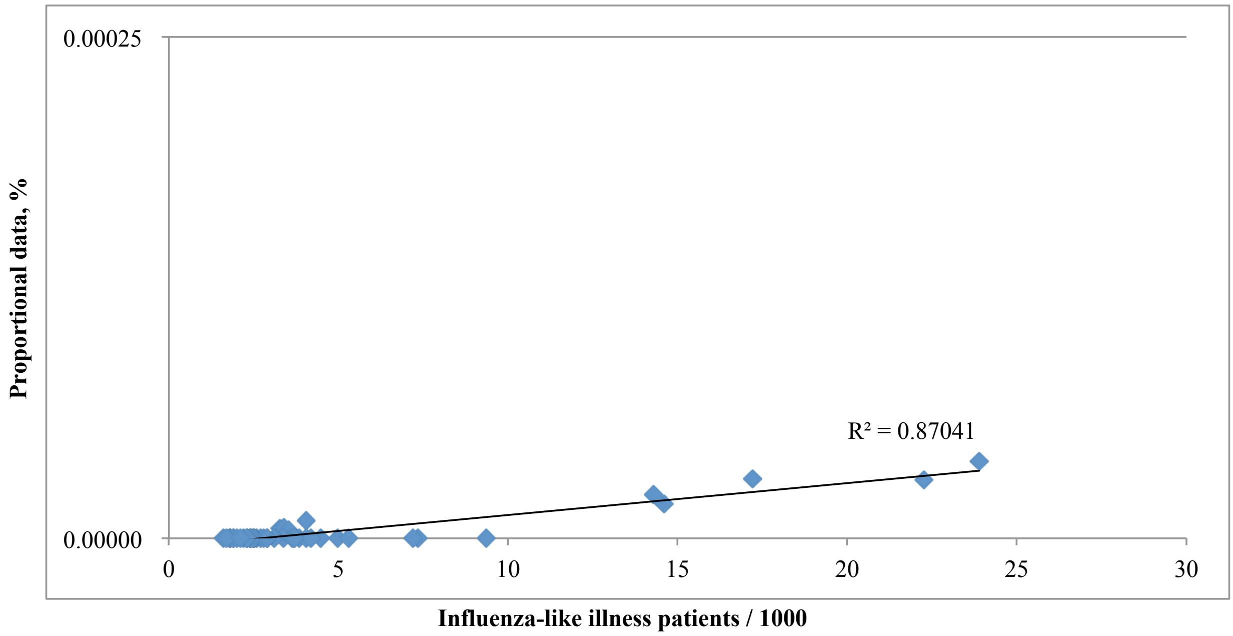

Cumulative query model 6 in validation set 1

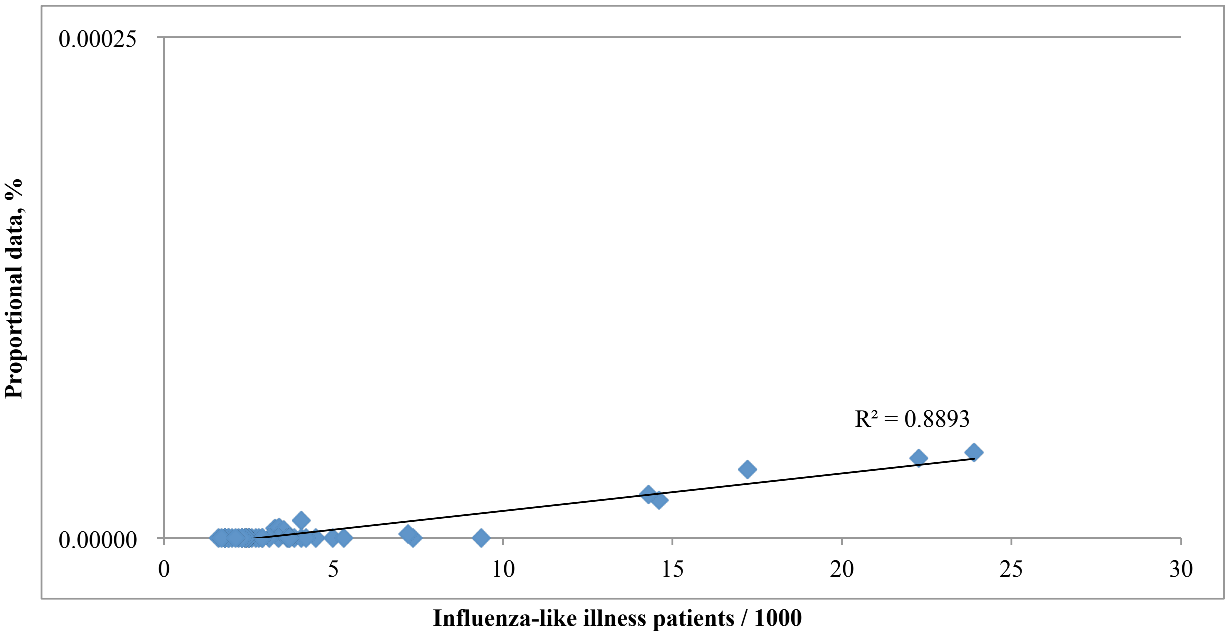

Cumulative query model 7 in validation set 1

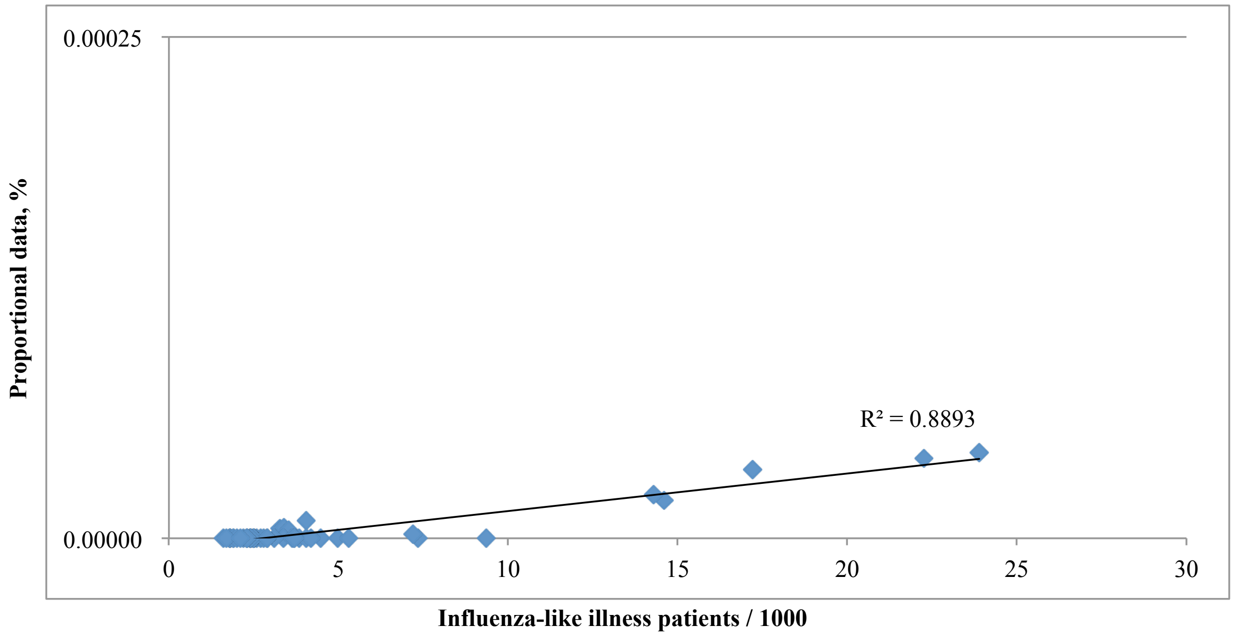

Cumulative query model 8 in validation set 1

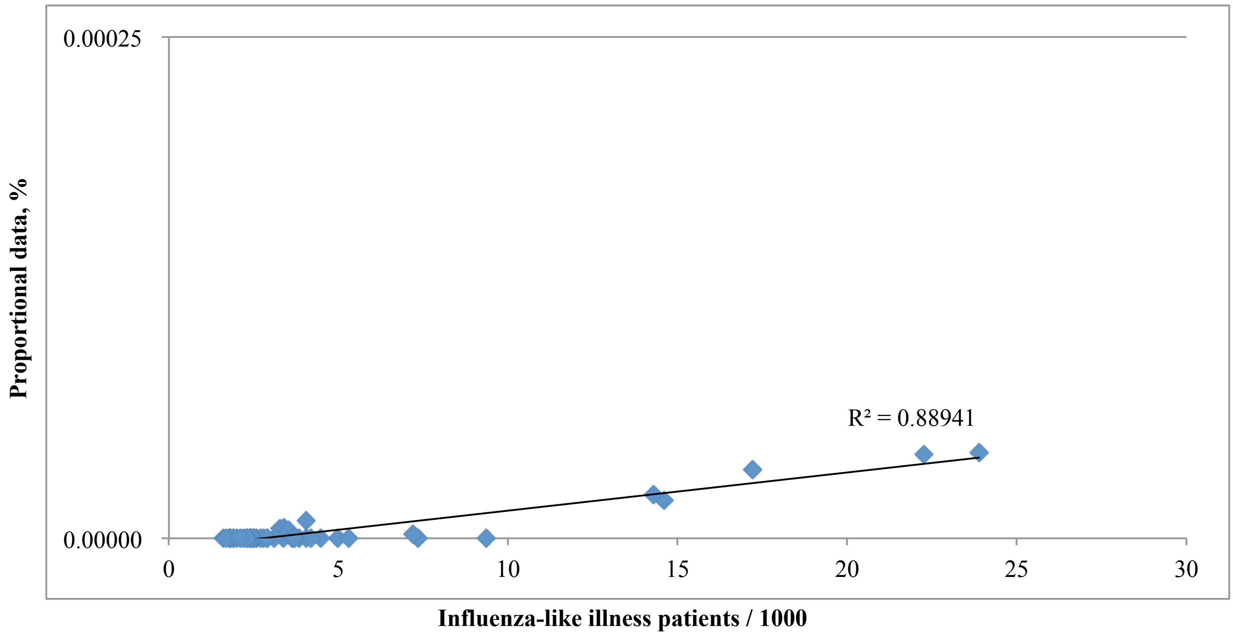

Cumulative query model 9 in validation set 1

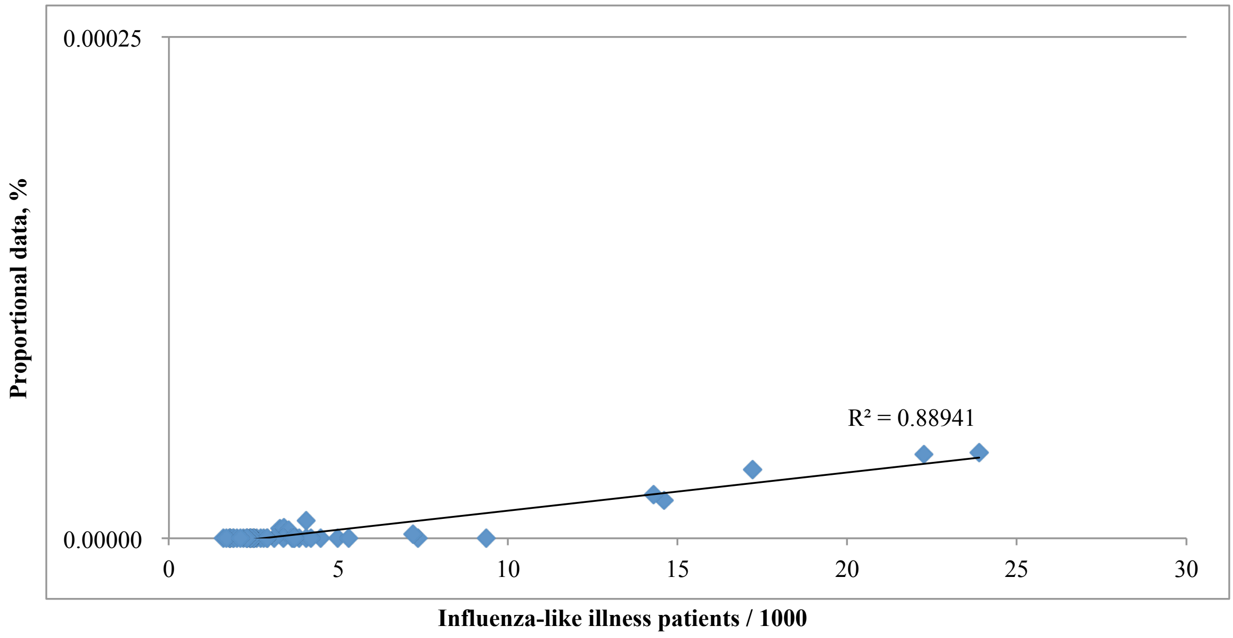

Cumulative query model 10 in validation set 1

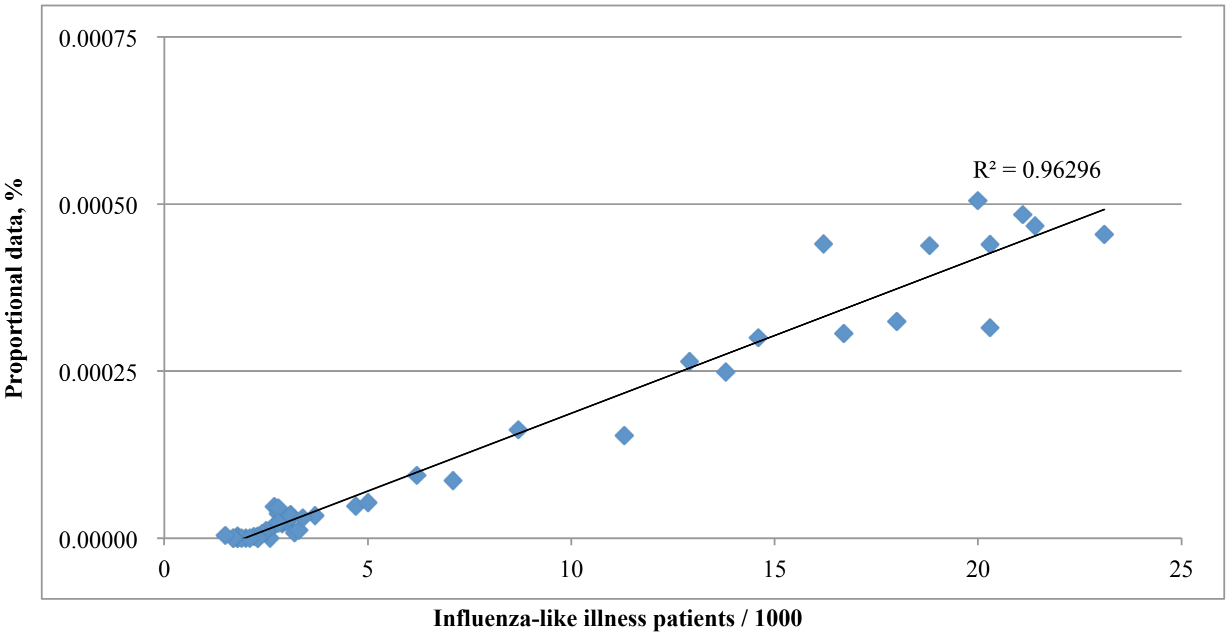

Cumulative query model 1 in validation set 2

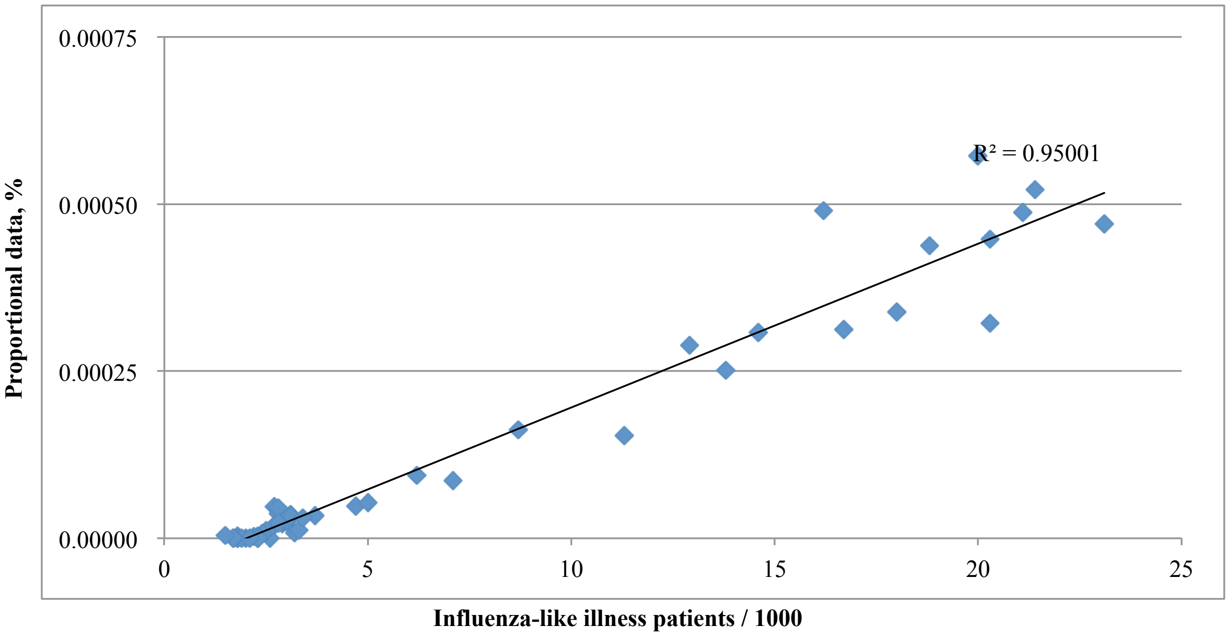

Cumulative query model 2 in validation set 2

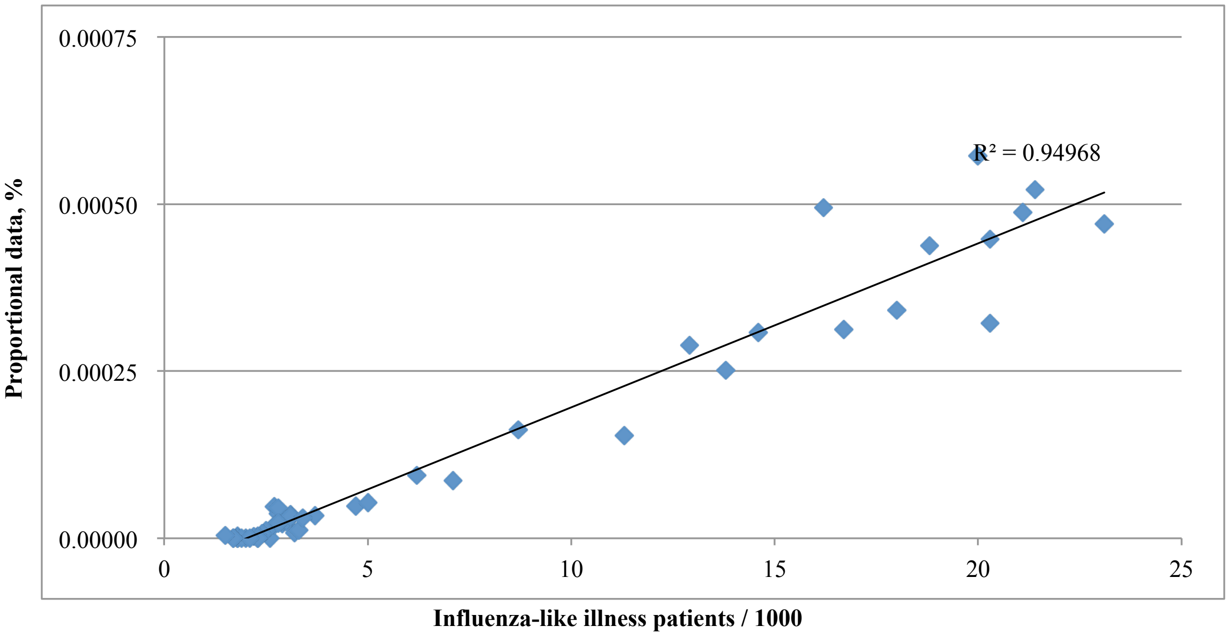

Cumulative query model 3 in validation set 2

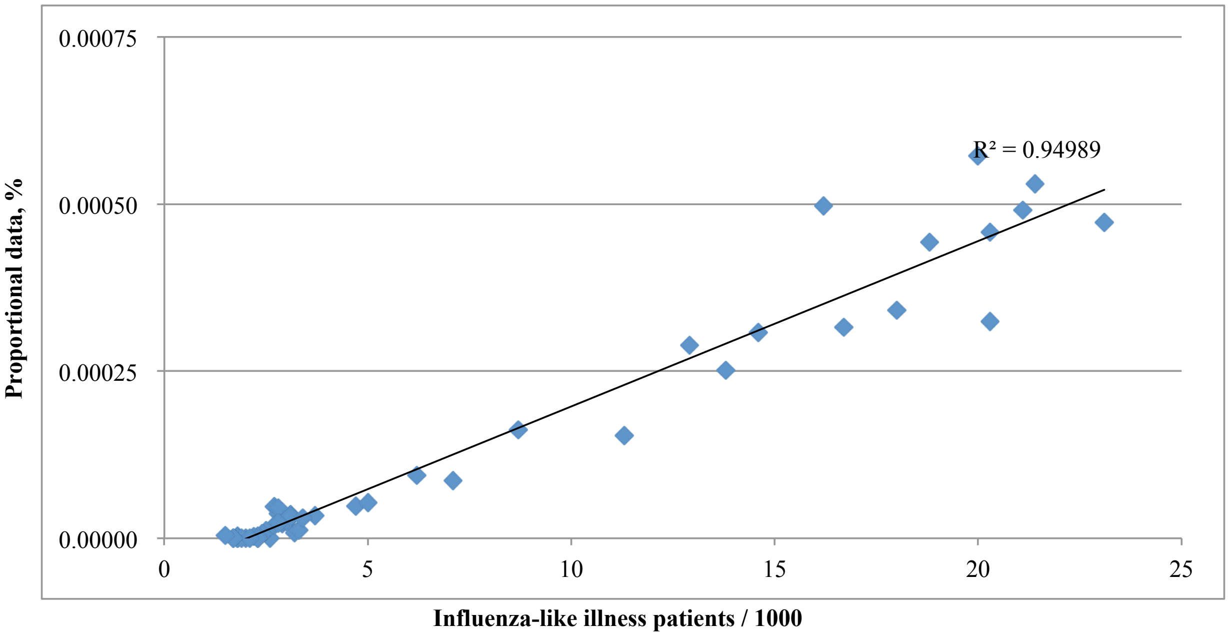

Cumulative query model 4 in validation set 2

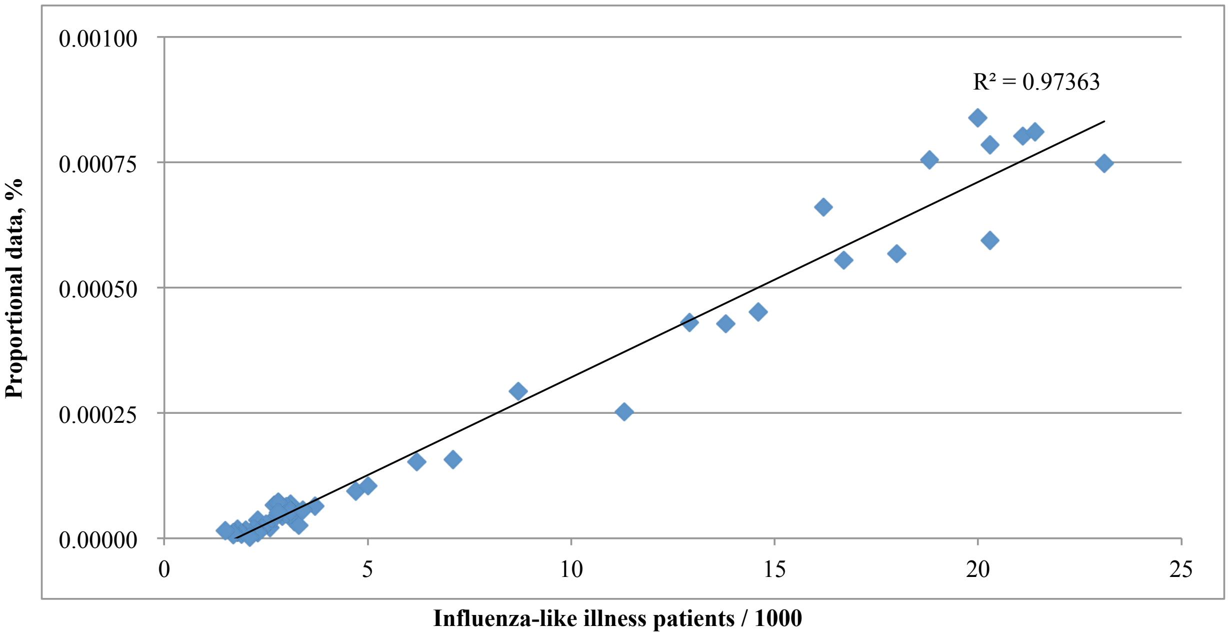

Cumulative query model 5 in validation set 2

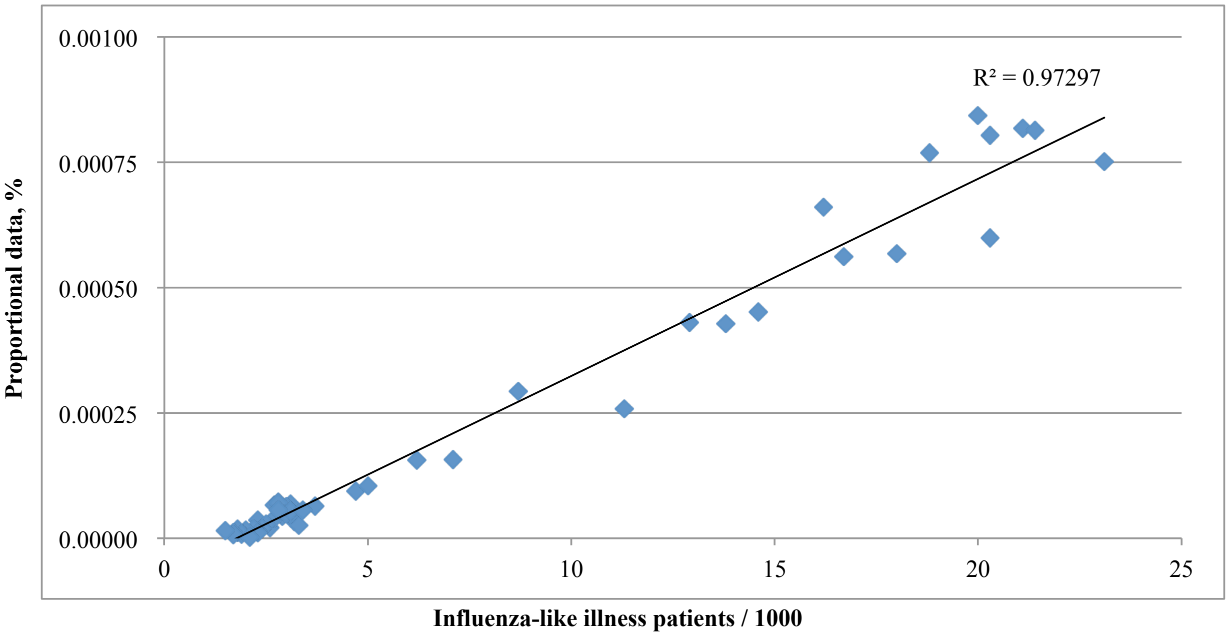

Cumulative query model 6 in validation set 2

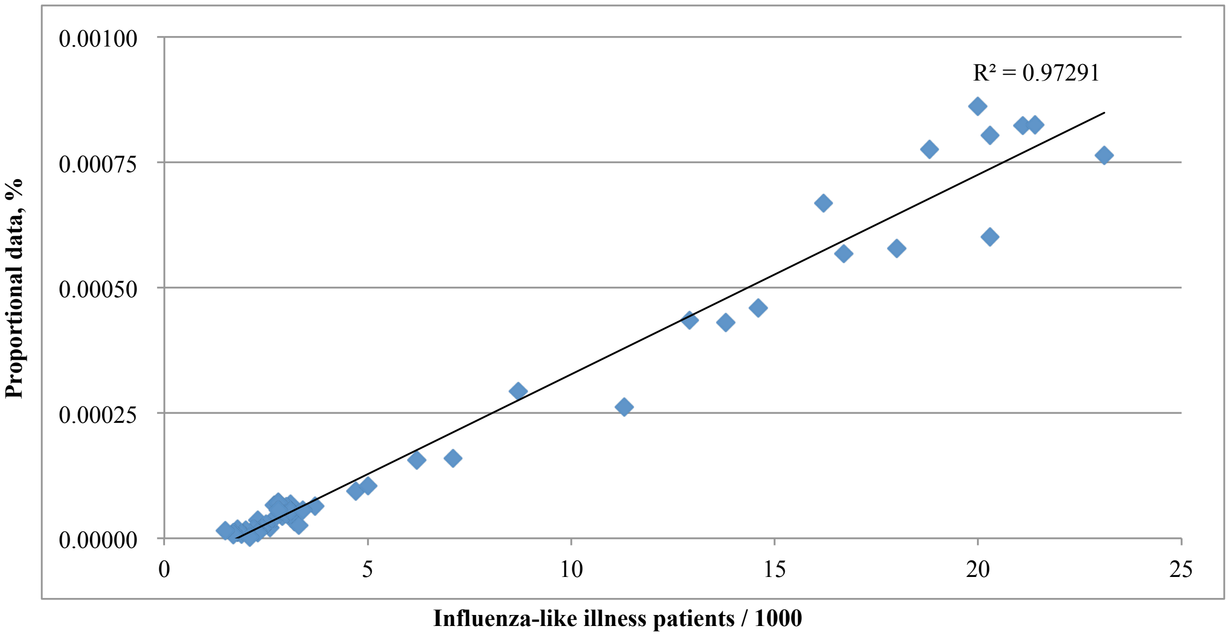

Cumulative query model 7 in validation set 2

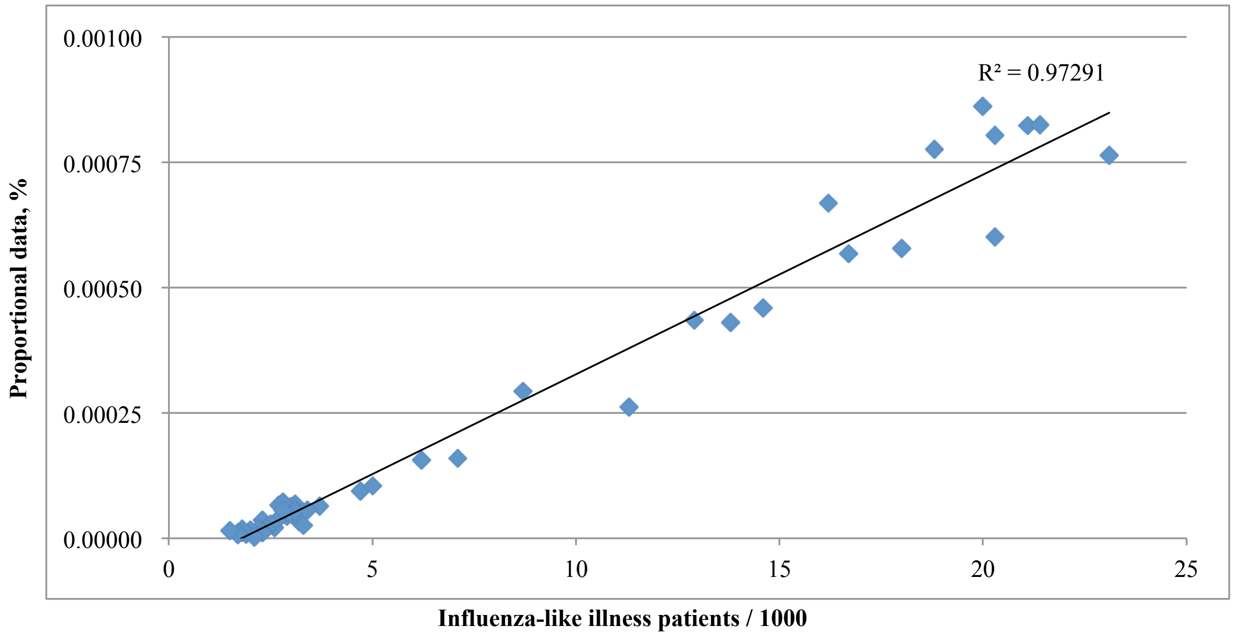

Cumulative query model 8 in validation set 2
