# Supplementary material for: Cumulative Query Method for Influenza Surveillance Using Search Engine Data
Source: J Med Internet Res. 2014 Dec 16;16(12):e289. doi: 10.2196/jmir.3680 (PMC4275481; doi:10.2196/jmir.3680)
Supplement: Supplementary file 5 [file jmir_v16i12e289_app5.pdf]

Correlation analysis between the DAUM data for combined queries and the KCDC[a] virologic data in development sets 1 and 2.

	Development set 1		Development set 2	
Order	Query	Correlation Coefficients[b]	Query	Correlation Coefficients[b]
1	Bad cold + Symptom	0.661	Bad cold + Symptom	0.892
2	Bad cold + Treatment	0.493	New flu + Sign	0.876
3	New flu (abbr.)[c]	0.464	New flu + Treatment	0.864
4	Flu + Vaccine	0.433	Tamiflu	0.84
5	Epidemic bad cold	0.427	New influenza + Symptom	0.823
6	New flu (abbr.)[c] + Vaccine	0.418	Swine flu + Symptom	0.821
7	Bad cold	0.411	New flu + Cough	0.821
8	New flu + Cough	0.408	New flu + Symptom	0.781
9	New flu + Vaccine	0.403	Bird flu + Symptom	0.779
10	New flu + fever	0.359	Bad cold + Treatment	0.762
11	Tamiflu + Symptom	0.355	Bird flu	0.745
12	H1N1 + Symptom	0.352	Influenza + Symptom	0.742
13	Epidemic bad cold + Symptom	0.346	Flu + Symptom	0.727
14	New flu + Treatment	0.345	Influenza (English)[d]	0.7
15	Bad cold + Sign	0.339	New influenza	0.697
16	Tamiflu + Vaccine	0.33	New flu (abbr.)[c]	0.615
17	Flu + Symptom	0.328	Swine flu	0.582
18	Tamiflu	0.323	Bird flu + Prevention	0.582
19	New flu + Symptom	0.321	Flu	0.568
20	New flu + Sore throat	0.313	New flu + fever	0.547
21	New flu + neck pain	0.293	Bird flu + Vaccine	0.537
22	New bad cold	0.287	Influenza + Prevention	0.521
23	Bird flu + Vaccine	0.282	New flu + Complication	0.502
24	Bird flu + Prevention	0.276	Bird flu + Decease	0.489
25	Tamiflu + Fever	0.274	Influenza	0.475
26			New flu + Prevention	0.46
27			Epidemic bad cold	0.452
28			Tamiflu (English)[d]	0.446
29			New flu + Sore throat	0.419
30			Bad cold + Decease	0.358
31			Epidemic bad cold + Symptom	0.358
32			Influenza + Decease	0.358
33			New flu + Mask	0.321

[a]KCDC : Korea Centers for Disease Control and Prevention

[b]*P*<.05.

[c]"(abbr).": abbreviation

[d]"(Enlish)": The query is originally submitted in English.

Correlation between cumulative query method n and KCDC virologic data in validation set 2.[a]

Cumulative Query Method	Correlation coefficients[b]
1	0.915[c]
2	0.915[c]
3	0.906[c]
4	0.916[c]
5	0.916[c]
6	0.916[c]
7	0.916[c]
8	0.885
9	0.886
10	0.886
11	0.774
12	0.789
13	0.789
14	0.79

[a]We selected the combined queries for which the correlation coefficients were 0.7 or higher and listed them in descending order. We then created a cumulative query model n representing the number of cumulative combined queries in descending order of the correlation coefficients.

[b] $P<.05$.

[c]Useful cumulative query model in the validation set was defined as having higher correlation coefficient than the highest correlation coefficient of single combined query in the same development set.
